# Vitamin D Deficiency in Human and Murine Sepsis*

**DOI:** 10.1097/CCM.0000000000002095

**Published:** 2017-01-18

**Authors:** Dhruv Parekh, Jaimin M. Patel, Aaron Scott, Sian Lax, Rachel C. A. Dancer, Vijay D’Souza, Hannah Greenwood, William D. Fraser, Fang Gao, Elizabeth Sapey, Gavin D. Perkins, David R. Thickett

**Affiliations:** 1 Centre for Translational Inflammation Research, Institute of Inflammation and Aging, University of Birmingham, Birmingham, United Kingdom.; 2 Warwick Clinical Trials Unit, Warwick Medical School, University of Warwick, Coventry, United Kingdom.; 3 Academic Department of Anesthesia, Critical Care, Resuscitation and Pain, Heart of England NHS Foundation Trust, Bordesley Green, Birmingham, United Kingdom.; 4 Norwich Medical School, University of East Anglia, Norwich, United Kingdom.

**Keywords:** cecal ligation and puncture, sepsis, vitamin D deficiency

## Abstract

**Objectives:**

Vitamin D deficiency has been implicated as a pathogenic factor in sepsis and ICU mortality but causality of these associations has not been demonstrated. To determine whether sepsis and severe sepsis are associated with vitamin D deficiency and to determine whether vitamin D deficiency influences the severity of sepsis.

**Design, Setting, and Patients:**

Sixty-one patients with sepsis and severe sepsis from two large U.K. hospitals and 20 healthy controls were recruited. Murine models of cecal ligation and puncture and intratracheal lipopolysaccharide were undertaken in normal and vitamin D deficient mice to address the issue of causality.

**Measurements and Main Results:**

Patients with severe sepsis had significantly lower concentrations of 25-hydroxyvitamin D_3_ than patients with either mild sepsis or age-matched healthy controls (15.7 vs 49.5 vs 66.5 nmol/L; *p* = 0.0001). 25-hydroxyvitamin D_3_ concentrations were significantly lower in patients who had positive microbiologic culture than those who were culture negative (*p* = 0.0023) as well as those who died within 30 days of hospital admission (*p* = 0.025). Vitamin D deficiency in murine sepsis was associated with increased peritoneal (*p* = 0.037), systemic (*p* = 0.019), and bronchoalveolar lavage (*p* = 0.011) quantitative bacterial culture. This was associated with reduced local expression of the cathelicidin-related antimicrobial peptide as well as evidence of defective macrophage phagocytosis (*p* = 0.029). In the intratracheal lipopolysaccharide model, 1,500 IU of intraperitoneal cholecalciferol treatment 6 hours postinjury reduced alveolar inflammation, cellular damage, and hypoxia.

**Conclusions:**

Vitamin D deficiency is common in severe sepsis. This appears to contribute to the development of the condition in clinically relevant murine models and approaches to correct vitamin D deficiency in patients with sepsis should be developed.

## Abstract 2

Supplemental Digital Content is available in the text.

Sepsis remains a common cause for hospital admission and is the commonest reason for admission to ICUs. Despite improvements in management of sepsis, the prevalence of sepsis continues to increase and is the leading cause of death in critically ill patients, affecting approximately 750,000 U.S. patients annually with a mortality rate of approximately 25% (1, 2).

Sepsis describes a complex clinical syndrome that results from a harmful or damaging host response to infection. A significant proportion of patients with sepsis go on to develop severe sepsis or septic shock (1, 2). Despite considerable research, there still remains a lack of targeted pharmacologic interventions to treat and improve outcomes from sepsis (3, 4).

Over 1 billion people worldwide are believed to have vitamin D deficiency (VDD) (5). Epidemiologic studies have demonstrated that 25-hydroxyvitamin D_3_ (25[OH]D_3_) concentrations are related to geography and season (6, 7). In a study of middle-aged adults in the United Kingdom, 40% had serum 25(OH)D_3_ concentrations above 30 ng/L (75 nmol/L) in the summer months but this decreased to less than 17% in the winter (8). The prevalence and mortality of sepsis is higher during the winter when 25(OH)D_3_ concentrations are lower (9).

Vitamin D metabolites have important pleiotropic effects on human immunity, acting as modulators of cells of the innate and adaptive system (10). Biologically active 1,25(OH)_2_D_3_ directly enhances signaling to increase antimicrobial peptides, cathelicidin (LL-37, its active form), and β-defensin by the innate immune system (11). Gram-positive bacteria, invasive pneumococcal disease, meningococcal disease, and group A streptococcal disease are more common when 25(OH)D_3_ concentrations are low (12).

VDD is associated with an increased risk of ICU admission and mortality in patients with pneumonia (13). Studies suggest VDD is common in critically ill patients and associated with adverse outcome (14, 15). Patients with sepsis who are not vitamin D sufficient (VDS) have an increased risk of mortality after critical care initiation (16). Recent meta-analyses support the association of VDD with increased susceptibility for severe infection, sepsis, and mortality in the critically ill (17, 18).

We believe that VDD is a determinant of the severity of sepsis because of effects on the host defense against infection. Previous studies of VDD have largely concentrated upon patients recruited within ICU. The prevalence and clinical significance in terms of outcomes of VDD in hospitalized mild sepsis outside the ICU is unknown.

Our aim was to determine the prevalence and severity of VDD in a cohort of sepsis patients both within ICU and in a medical admissions unit (MAU) environment as soon as possible after hospital admission. We used murine sepsis models to explore the mechanistic link between preinjurious VDD and sepsis. Finally, we undertook studies in intratracheal lipopolysaccharide (IT-LPS)-treated VDD mice to demonstrate whether rescue therapy with vitamin D can attenuate inflammatory lung damage and dysregulated apoptosis associated with VDD (19).

## METHODS

Detailed methods are available in the **online data supplement** (Supplemental Digital Content 1, http://links.lww.com/CCM/C170).

### Study Participants

Patients were recruited from the acute MAUs (AMUs) and ICUs at two University Hospitals (Heart of England NHS Foundation Trust and the University Hospital Birmingham NHS Foundation Trust) between September 2012 and October 2014 as part of an observational sepsis study. Healthy elderly volunteers were recruited from a registry at the University of Birmingham and provided a cohort of healthy elderly individuals that would act as age-matched controls for the sepsis patients.

### Ethical Approvals

All patients and healthy volunteers provided informed written consent. In circumstances where patients were unable to provide consent, a legal representative (personal or designated consultee) provided assent. Retrospective consent was sought where possible, when patients regained the ability to consent. This study received the appropriate ethical committee and local governance approvals (research ethics committee 11/YH0270).

### Inclusion Criteria

Age greater than 18 years; documented new proven or suspected infection, and the presence of any two of the signs and symptoms of infection (WCC, > 11 or < 4 × 10^9^/L; temperature, > 38°C or < 36°C; heart rate, > 90 per beats/min; or respiratory rate, > 20 per minute) for less than 24 hours. Patients were categorized as sepsis or severe sepsis, according to the presence of one or more organ failure at admission (20).

### Exclusion Criteria

Recent chemotherapy, chronic steroid use, or use of other immunosuppressant drugs.

### Laboratory Methods

25(OH)D_3_ was measured by tandem mass spectrometry. The assay is calibrated using National Institute of Standards and Technology aligned material, achieving certification within Vitamin D External Quality Assessment Scheme, and described in detail previously (21). 25(OH)D_3_ concentrations below 50 nmol/L (20 ng/mL) were regarded as deficient. 25(OH)D_3_ concentrations between 50 and 75 nmol/L (30 ng/mL) were regarded as insufficient, with concentrations above 75 nmol/L (30 ng/mL) designated sufficient (22).

### Animal Materials and Methods

#### Induction of VDD.

Male wild-type (WT) C57Bl/6 mice once weaned were made VDD by feeding them a VDD chow (TD 89123: Harlan, Madison, WI) or maintained on normal chow for 6 weeks as a control.

#### Murine Models of Sepsis and Lung Injury.

Cecal ligation and puncture (CLP) and intratracheal instillation of LPS were performed as described previously (23) and in the online data supplement (Supplemental Digital Content 1, http://links.lww.com/CCM/C170). Cell counts, protein permeability index (PPI), quantitative bacterial culture, cathelicidin-related antimicrobial peptide (CRAMP) levels in peritoneal lavage fluid (PLF), blood and bronchoalveolar lavage fluid (BALF), and peritoneal macrophage phagocytosis were compared between VDD and VDS mice in the CLP model. BALF cell counts, PPI, receptor for advanced glycation end-products (RAGE), and oxygen saturations were compared in the IT-LPS model.

### Statistics

Initial power calculations for eight animals in each arm for the CLP experiment were based upon preliminary data to detect a change in lung PPI of 20% between VDD and VDS mice and six animals per arm to see a treatment effect on BALF neutrophils of 15% in the IT-LPS experiment. CLP experiments were performed in batches of four mice at different time points. Uneven numbers account for a failure in the experiment. Data were analyzed using SPSS for Windows 16.0 (SPSS, Chicago, IL). Data were tested for normality using a Shapiro-Wilks test with parametric data analyzed using unpaired *t* tests and a Mann-Whitney *U* test for nonparametric data. Data are expressed as mean (sd) unless otherwise indicated. A chi-square or Fisher exact test was used to compare proportions. A two-tailed *p* value of less than 0.05 was considered significant.

## RESULTS

### Patient Cohorts

#### Sepsis Patients.

We enrolled 61 patients with sepsis—20 had mild sepsis and 41 had severe sepsis. Twelve patients were enrolled in the ICU and 49 were enrolled in AMUs. Two patients were transferred from AMUs to ICU postrecruitment. The etiology of sepsis was predominantly community-acquired pneumonia with a similar range of causes between sepsis and severe sepsis. As expected severity scores Acute Physiology and Chronic Health Evaluation (APACHE) II and SOFA score were significantly greater in severe sepsis than mild sepsis (**Table 1**)

**TABLE 1.**
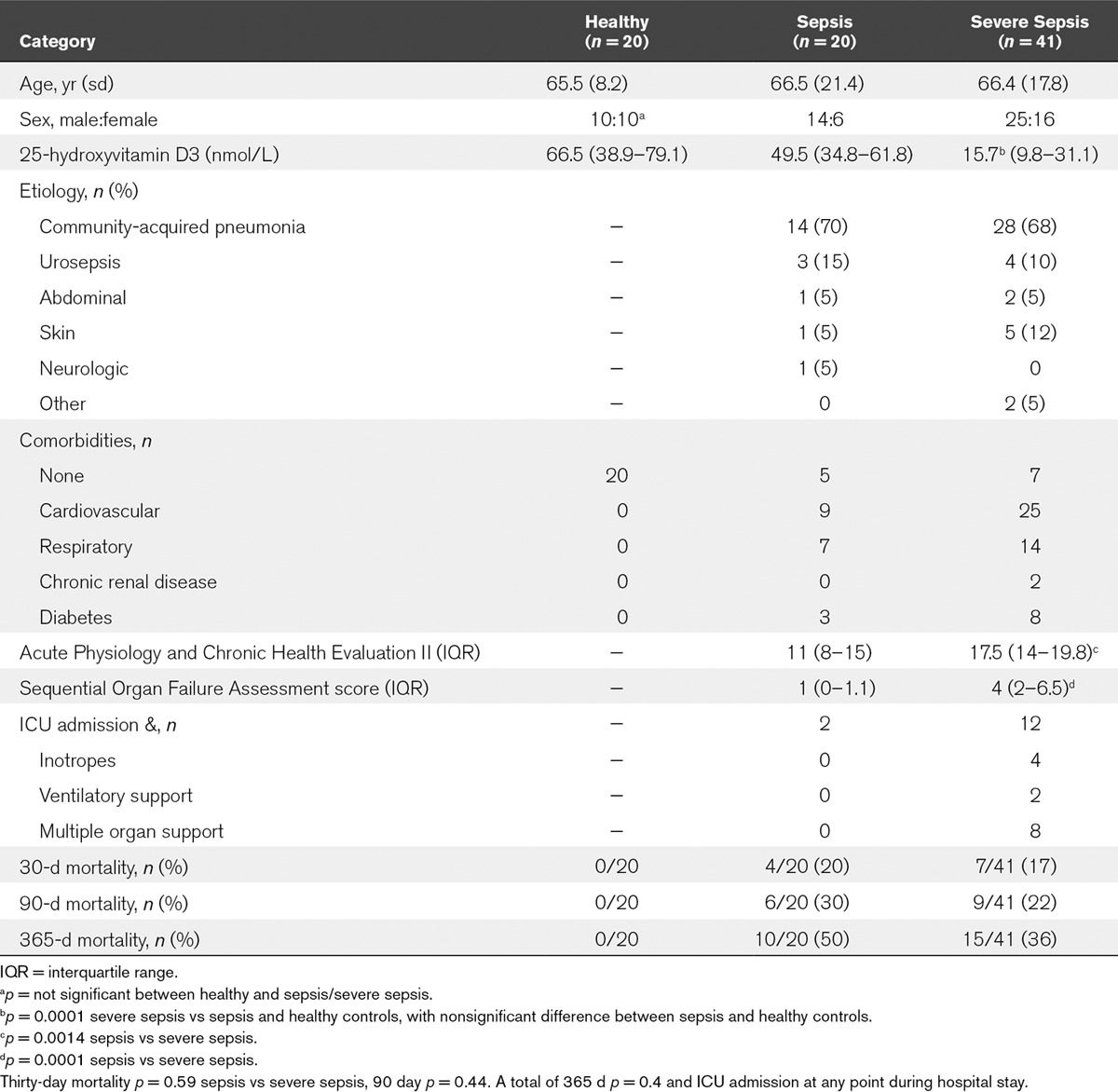
Demographics and Severity of Patient Cohorts/Volunteers

#### Healthy Donors.

Blood from 20 healthy elderly donors were obtained—volunteers had no evidence of significant acute or chronic disease, normal spirometry, and were medication free. There were no significant differences in age or sex distribution between the healthy cohort, sepsis patients, and severe sepsis patients (Table 1).

### 25(OH)D_3_ Concentrations Are Lower in Patients With Severe Sepsis Compared With Sepsis and Healthy Controls

Of 61 patients, 41 met the criteria for severe sepsis with 14 requiring critical care at some point during their admission. Patients with severe sepsis had significantly lower 25(OH)D_3_ concentrations than either sepsis patients or controls (15.7 vs 49.5 vs 66.5 nmol/L; *p* = 0.0001). In contrast, patients with sepsis did not have significantly lower concentrations of 25(OH)D_3_ than healthy controls (Table 1) (**online supplement Fig. 1**, Supplemental Digital Content 1, http://links.lww.com/CCM/C170).

### 25(OH)D_3_ Concentrations Are Lower in Sepsis Patients With Positive Microbial Cultures

Twenty-two patients (36%) had positive cultures (blood/urine/sputum/BALF) for bacterial growth from samples taken as part of their clinical workup. Median 25(OH)D_3_ concentrations were significantly lower in patients who were culture positive (16.5 nmol/L) compared with culture negative patients (35.5 nmol/L; *p* = 0.0023) (**online supplement Fig. 2**, Supplemental Digital Content 1, http://links.lww.com/CCM/C170).

**Figure 1.**
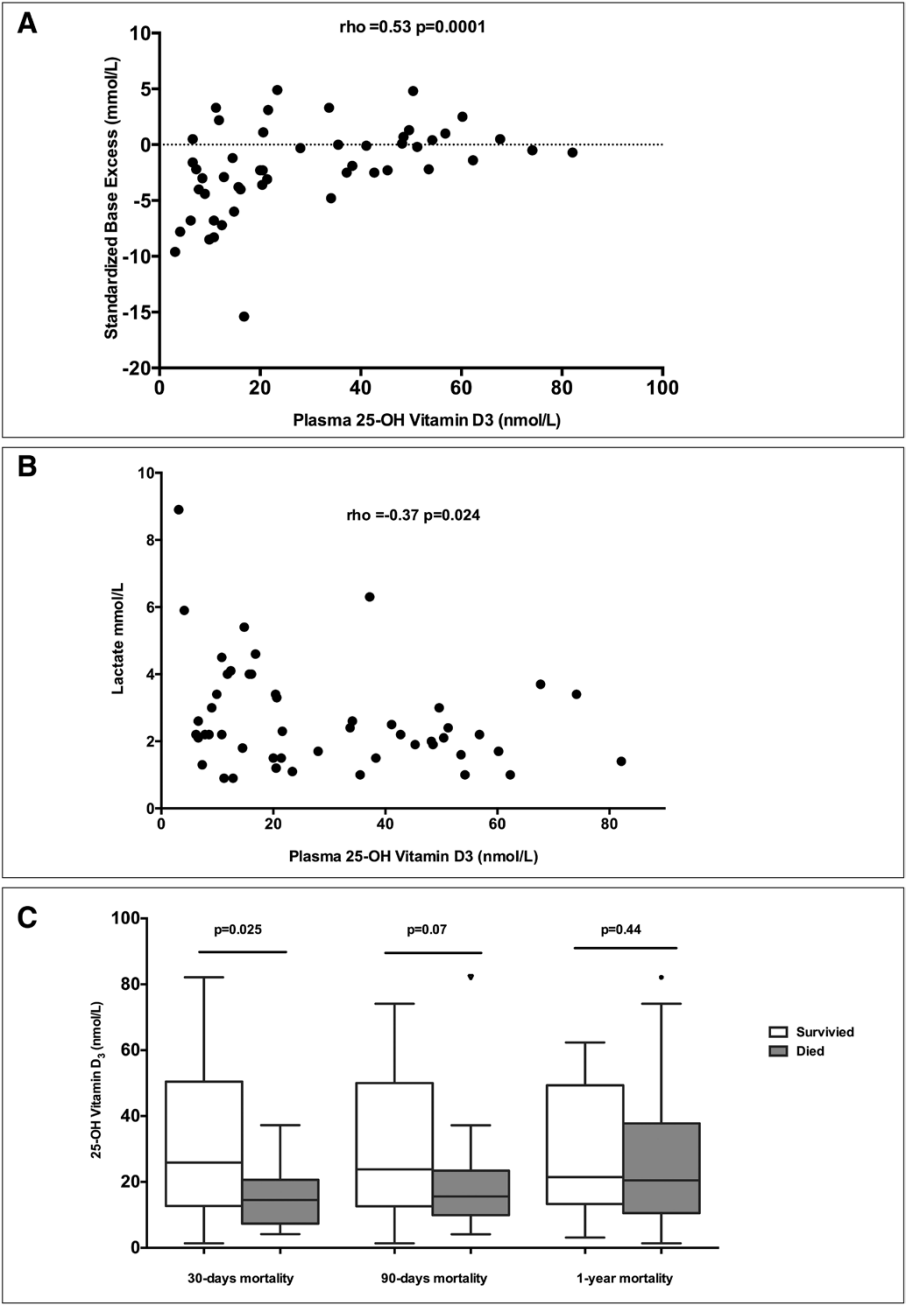
Regression plots for plasma 25-hydroxyvitamin D_3_ (25[OH]D_3_) and **A**, standardized base excess and **B**, blood lactate at admission. Regression was performed using Spearman’s rho for nonparametric data. **C**, Plasma 25(OH)D_3_ between survivors and nonsurvivors at 30 d, 90 d, and 1 yr. *Box* and *whisker plots* with median and Tukey’s distribution.

There was an inverse relationship between 25(OH)D_3_ at baseline and standardized base excess and blood lactate (mmol/L) (**Fig. 1**, ***A*** and ***B***). There was no relationship between 25(OH)D_3_ and age, sex, ethnicity, or the severity scores Sequential Organ Failure Assessment (SOFA) or APACHE II (data not shown).

### 25(OH)D_3_ Concentrations Are Lower in Patients Who Die Within 30 Days of Admission Than Survivors

Previous studies have suggested 25(OH)D_3_ concentrations are associated with mortality in ICU patients. In our whole cohort, there was a significant difference in median 25(OH)D_3_ concentrations between survivors (25.9 nmol/L) and nonsurvivors (14.5 nmol/L; *p* = 0.025) at 30 (11/61) days. This effect was not significant for 90-day mortality (15/61) or 1-year mortality (25/61) (**Fig. 1*C***)

### Severe VDD Is Associated With Increased Risk of Death in Patients With Sepsis

We have previously reported increased adverse postoperative inflammation and adverse events in patients undergoing esophagectomy who had severe deficiency before the operation (25[OH]D_3_, < 20 nmol/L) (19). In line with these data, sepsis patients with 25(OH)D_3_ concentrations below 20 nmol/L had a significant increased risk of 30-day mortality. Fisher exact test was significant at *p* value equal to 0.02 giving a relative risk of 4.71 (95% CI, 1.089–20.42).

## MURINE STUDIES

### Murine Vitamin D Status

VDD was successfully established in WT C57BL/6 mice fed a deficient diet compared with a VDS diet (**online supplement Table 1**, Supplemental Digital Content 1, http://links.lww.com/CCM/C170). 25(OH)D_3_ concentrations in the mice were equivalent to those seen in patients who died from severe sepsis. Deficiency did not result in a significant effect on serum calcium but was associated within reduced circulating bioactive 1,25(OH)_2_D_3_.

### VDD Is Associated With Increased Peritoneal/Systemic Bacteremia and Alveolar Bacterial Translocation After CLP

**Figure 2.**
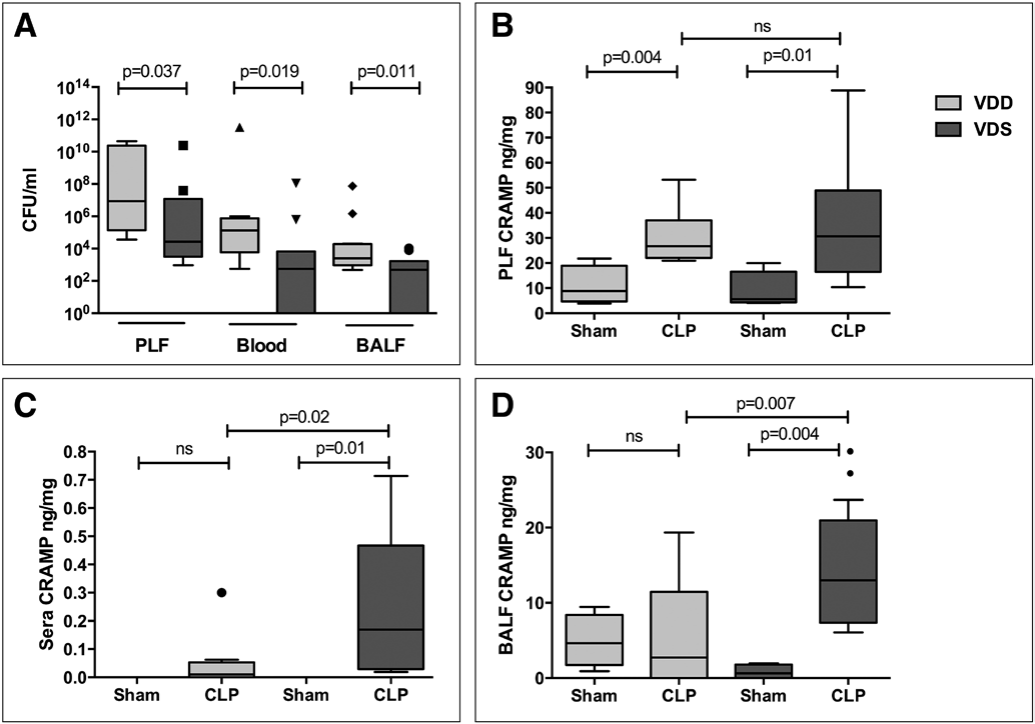
**A**, Effect of vitamin D deficient (VDD) on bacterial load in peritoneal lavage fluid (PLF), blood, and bronchoalveolar lavage fluid (BALF). Data presented as *box* and *whisker plots* with median and Tukey’s distribution, logarithmic scale to allow graphical representation. VDD *n* = 12; vitamin D sufficient (VDS) *n* = 11. Cathelicidin-related antimicrobial peptide (CRAMP) (murine cathelicidin) expression in **B**, PLF; **C**, serum; and **D**, BALF. *Box* and *whisker plots* with medians and Tukey’s distribution. VDD *n* = 12; VDS *n* = 11. Sham *n* = 4 per group. CRAMP was undetectable in sham-treated sera. CFU = colony forming units, CLP = cecal ligation and puncture, NS = not significant.

VDD mice had a significantly higher bacterial load compared with VDS mice in all three compartments (peritoneal, blood, and alveolar) 16 hours after CLP (**Fig. 2*A***). In sham experiments, there was an absence of bacteria as measured by colony forming units per milliliter in all three compartments confirming sterile procedure and surgery (data not shown).

### CRAMP Is Reduced in VDD Mice

CRAMP has been widely identified as a vitamin D-dependent antimicrobial peptide that binds bacteria. Cathelicidin rapidly destroys the lipoprotein membranes of microbes enveloped in phagosomes after fusion with lysosomes in macrophages (24).

The CLP procedure increases CRAMP concentrations significantly in PLF, serum, and BALF in VDS mice. However, significantly lower concentrations were observed in VDD mice supporting the observation that VDD mice have reduced antimicrobial capacity (**Fig. 2**, ***B***–***D***).

### CLP Does not Induce Alveolar Neutrophilia but Does Increase PPI, Which Is More Pronounced in VDD

Little-to-no cellular recruitment into the alveolar compartment was observed at this time point; however, there was evidence of a mild increase in BALF PPI, suggesting early alveolar epithelial leak. This was significantly higher in VDD mice when compared with VDS mice (median, 3.30 [interquartile range (IQR), 2.69–4.64] vs 2.09 [IQR, 1.82–2.90]; *p* = 0.014) (**online supplement Fig. 3**, Supplemental Digital Content 1, http://links.lww.com/CCM/C170).

### VDD Is Associated With Increased Cellular Inflammation in the Peritoneum

After CLP there was significant cellular recruitment in PLF. As major players of the acute inflammatory response, neutrophils and F4/80+ macrophages were enumerated within the peritoneal cavity. Significantly more neutrophils and F4/80+ macrophages were observed in VDD compared with VDS mice after CLP, with the neutrophil-to-macrophage ratio similar between both groups indicating a global increase in inflammatory mediators. PLF PPI was also significantly increased in VDD mice (median, 46.86 [IQR, 28.17–58.33] vs 29.81 [14.81–54.52]; *p* = 0.06) also indicative of more vascular damage in VDD mice after CLP (**online supplement Fig. 4**, Supplemental Digital Content 1, http://links.lww.com/CCM/C170).

### VDD Is Associated With Dysregulated Neutrophil Apoptosis and Impaired Peritoneal Macrophage Phagocytosis of Bacteria After CLP

**Figure 3.**
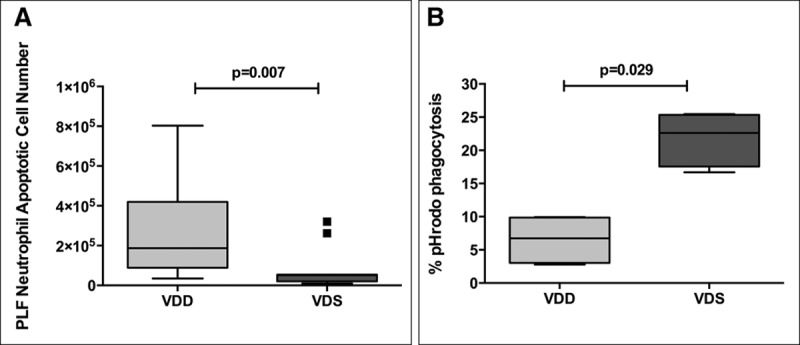
**A**, Peritoneal lavage fluid (PLF) neutrophil apoptosis. *Box* and *whisker plot* with median and Tukey’s distribution, vitamin D deficient (VDD) *n* = 12; vitamin D sufficient (VDS) *n* = 11. **B**, PLF macrophage phagocytosis of pHrodo-labeled *E. coli* bacteria. *Box* and *whisker plot* with median and Tukey’s distribution, Mann-Whitney test. VDD *n* = 4; VDS *n* = 4.

There was a significant increase in the number of apoptotic neutrophils in VDD compared with VDS PLF (median, 1.87 × 10^5^ [IQR, 0.89–4.19 × 10^5^] vs 0.51 × 10^5^ [IQR, 0.20–0.54 × 10^5^]; *p* = 0.007) (**Fig. 3*A***), suggesting dysregulated neutrophil apoptosis and clearance of dying cells. To determine whether the increased bacteremia and/or accumulation of apoptotic neutrophils observed in VDD mice was due to impaired clearance by peritoneal macrophages, we assessed bacterial phagocytosis after CLP. Ex vivo phagocytosis of pHrodo-labeled *Escherichia coliform* bacteria was significantly reduced in F4/80+ macrophages isolated from PLF of VDD compared with VDS mice after CLP (median, 6.89% [IQR, 3.12–9.87] vs 21.12% [IQR, 17.56–24.29]; *p* = 0.029) (**Fig. 3*B***).

### Intraperitoneal (IP) Liquid Cholecalciferol (Vigantol) Rescue Therapy Attenuates Vitamin D-Related Inflammatory Damage Even When Given 6 Hours After IT-LPS Challenge

In the United Kingdom, we can only use the CLP model of early sepsis due to home office animal license rules. To assess whether rescue therapy with vitamin D was effective postinjury, we studied our well-characterized IT-LPS challenge model, which we have previously reported results in exaggerated inflammation in VDD mice (23). Animals were administered 1,500 IU (75 µL) of cholecalciferol (Vigantol; Merck Serono GmbH, Darmstadt, Germany) or phosphate buffered saline control IP injection 6 hours postinjury and killed after 48 hours.

**Figure 4.**
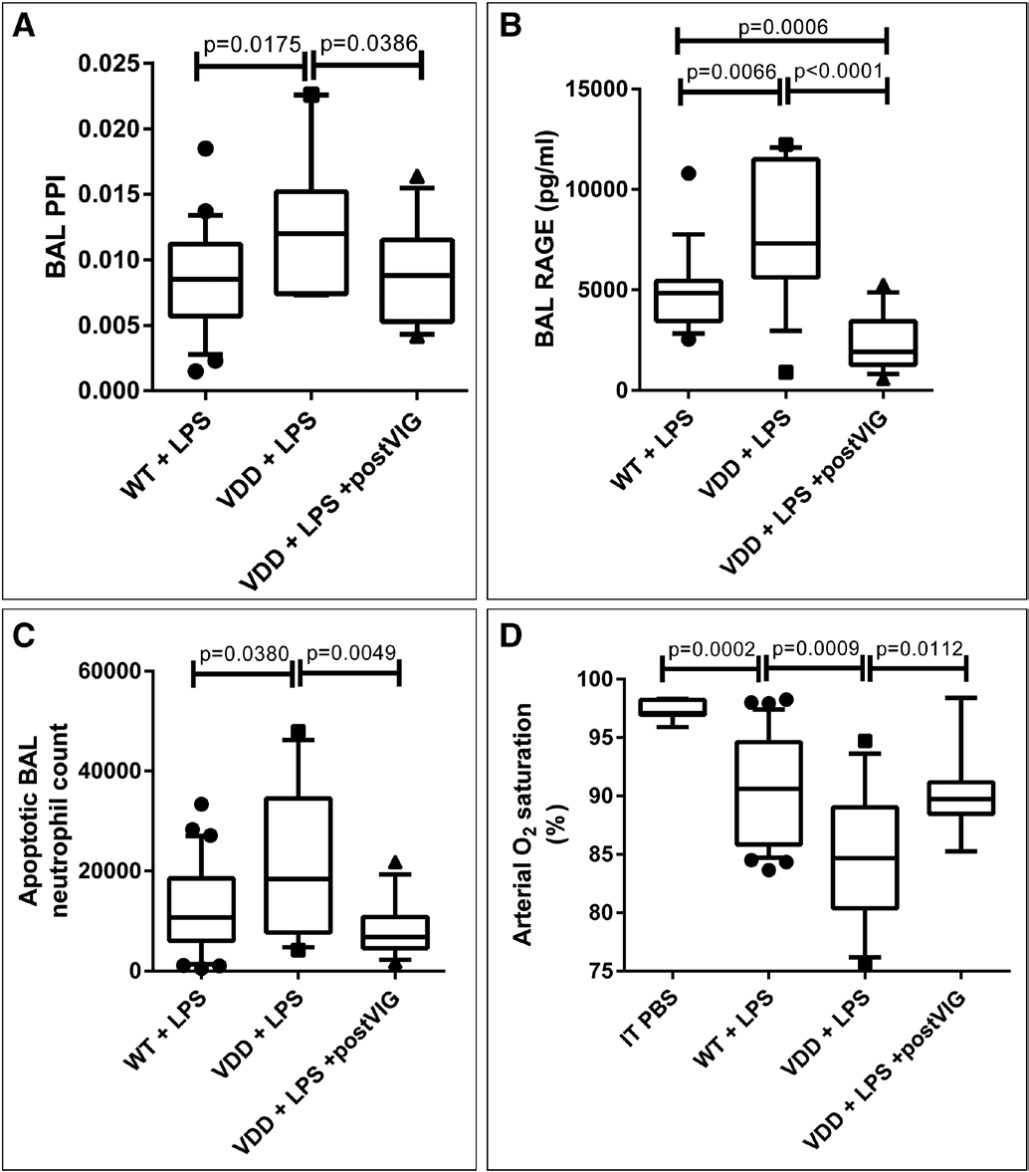
Effect of intraperitoneal liquid cholecalciferol (Vigantol) rescue therapy upon (**A**) bronchoalveolar lavage fluid protein permeability index (BAL PPI); **B**, BAL receptor for advanced glycation end-products (BAL RAGE); **C**, BALF total apoptotic cell count, and **D**, arterial oxygen saturation in wild-type (WT) vitamin D sufficient mice given intratracheal lipopolysaccharide (IT-LPS), vitamin D deficient (VDD) mice given IT-LPS, and VDD mice given IT-LPS and 1,500 IU unit rescue therapy with cholecalciferol (postVIG) 6 hr postinjury. Mice were killed at 48 hr post-IT-LPS (*n* = 6 per arm).

Vigantol administration restored 25(OH)D_3_ concentrations in VDD mice to those similar to WT, which was sufficient to normalize the lung injury post- IT-LPS (**online supplemental Fig. 5**, Supplemental Digital Content 1, http://links.lww.com/CCM/C170). Vigantol treatment 6 hours post-IT-LPS reduced BALF PPI, RAGE (a marker of alveolar epithelial damage), and normalized BALF neutrophil apoptosis (**Fig. 4**, ***A***–***C***). In addition, Vigantol attenuated the exaggerated decrease in oxygen saturations seen in this model with VDD mice (**Fig. 4*D***).

## DISCUSSION

We have confirmed, in a cohort of hospitalized patients with sepsis, that VDD is common, severe, and is associated with disease severity, bacterial positive culture, and 30-day mortality. To demonstrate causation of VDD as a driver of sepsis severity, our CLP mouse studies demonstrated exaggerated bacterial growth both locally and systemically, increased cellular inflammation, and dysregulated accumulation of apoptotic neutrophils in VDD mice. Using the IT-LPS challenge model, we demonstrate that the novel administration of IP cholecalciferol is an effective postinjury therapy when given 6 hours postinjury.

We enrolled a mixed population of both mild and severe sepsis patients. VDD was both common and severe in patients with severe sepsis. 25(OH)D_3_ concentrations were lower in patients who died than survived as well as patients who grew culture positive bacterial specimens. Additionally, clinical markers of sepsis severity (lactate, metabolic acidosis) were associated with lower levels of 25(OH)D_3_ suggesting perhaps these measures could reflect a VDD population in sepsis. A criticism often levelled at observational studies, such as ours, is whether the VDD is a marker of critical illness or a mechanistic driver. Two recent meta-analyses of observational studies have confirmed a significant association between vitamin D status and susceptibility to sepsis (18), rates of infection, and 30-day mortality (17). Our findings are concordant with observational studies that have demonstrated that low vitamin D status upon admission is associated with sepsis (16), bacteremia (25), and acute respiratory distress syndrome (26, 27).

The murine studies sought to establish whether inducing VDD by diet before injury in mice leads to exaggerated sepsis and enhanced cellular inflammation/dysfunction. We successfully established severe deficiency in the mice, with concentrations of 25(OH)D_3_ similar to those who died from sepsis in our clinical cohort. This deficiency was reflected also in reduced circulating 1,25(OH)_2_D_3_, the bioactive form of vitamin D. In contrast, our WT mice had 25(OH)D_3_ and 1,25 (OH)_2_D_3_ concentrations similar to our mild sepsis patient population.

In the clinically relevant CLP model of early sepsis, VDD was associated with exaggerated bacterial growth in the peritoneal cavity, elevated systemic bacteremia as well as increased bacterial translocation to the alveolar compartment. This was associated with abnormal protein permeability of the peritoneal and alveolar capillary barrier. In the PLF, there was exaggerated cellular inflammation in VDD mice with evidence of impaired antibacterial responses in terms of CRAMP release and the ability of peritoneal macrophages to phagocytose *E. coli*. These cellular changes resulted in increased accumulation of apoptotic neutrophils in the PLF. Previous animal studies have shown a benefit of 1,25(OH)_2_D_3_ on sepsis-induced coagulopathy in rats (28) and our CRAMP results confirm findings by others of decreased antimicrobial peptide in VDD in sepsis and critical illness (29, 30). Our study is the first to report VDD as a predeterminant of sepsis and decreased macrophage phagocytosis in a relevant murine model. These data support our hypothesis that VDD is mechanistically important in driving sepsis and led us to the question of whether treating deficiency postinjury would be an effective therapy.

In the United Kingdom, the regulatory framework for animal experiments dictated that we could not keep VDD animals alive post-CLP for more than 16 hours because of serious adverse events so we were only able to model early sepsis using this technique. Our group has recently shown a detrimental effect of VDD with exaggerated lung injury, dysregulated cellular inflammation, and apoptosis, which manifested as reduced oxygenation in an IT-LPS direct murine lung injury model 48 hours after injury (19). We, therefore, used our IT-LPS model to test whether postinjury treatment of VDD mice attenuated the effects of VDD upon inflammatory injury.

Traditionally, vitamin D supplements have been given by mouth, intramuscular injection (cholecalciferol and ergocalciferol), or by IV infusion (calcitriol) with mixed results potentially due to poor absorption from muscle or the gut or a short IV half-life (31–33). We elected to test the effect of IP administration of 1,500 IU cholecalciferol liquid as a novel route to restore VDS—a dose that proved effective in restoring 25(OH)D_3_ concentrations back to those seen in WT mice. Postinjury cholecalciferol therapy was effective in reducing the exaggerated cellular inflammation, alveolar epithelial damage as measured by PPI and RAGE release, and reduced hypoxia (oxygen saturations) when given 6 hours after the insult supporting IP administration of cholecalciferol as a novel potential route of administration in patients as well as evidence that restoration of vitamin D levels may reduce inflammation with physiologic benefit.

This study has limitations. First, patients were recruited up to 48 hours after hospital admission, so it is possible that the 25(OH)D_3_ concentrations seen reflected changes associated with sepsis rather than the cause. Second, this is a retrospective study of a small number of patients that could not control for patient comorbidities. The effects of sepsis and critical illness on the vitamin D metabolome are unknown and this complex interplay needs prospective large-scale studies that consider other preinsult comorbidities, chronic illness, nutritional status, and other confounders. It was for this reason we did the murine studies. In our CLP model, we studied early sepsis due to restrictions from the animal ethics committee. This meant that our animals had limited alveolar damage, which was why we undertook additional studies in the IT-LPS model. Although the VDD induced by diet design investigated whether pre-existing VDD is causal and a mechanistic driver to the severity of sepsis rather than the consequence of the sepsis insult in the murine model, it may not wholly explain the findings of the human study due to the lack of vitamin D status before sepsis and its effects on vitamin D status as discussed above. Finally, we have shown the effects of VDD in only two models of murine lung injury. Further work in other models related to sepsis particularly experimental pneumonia need to be undertaken.

In conclusion, we suggest that therapies aimed at restoring VDS in patients at risk of deficiency when they are admitted to hospital need to be developed to try and prevent the increasing healthcare burden of sepsis patients. Key to this will be establishing appropriate dosing regimens for vitamin D replacement in the critically ill patients both within and outside the ICU.

## ACKNOWLEDGMENTS

We thank Sister Teresa Melody for her assistance in recruiting patients with sepsis.

## Supplementary Material
