# Supplementary material for: Vitamin D Deficiency in Human and Murine Sepsis*
Source: Crit Care Med. 2017 Jan 18;45(2):282–9. doi: 10.1097/CCM.0000000000002095 (PMC5228606; doi:10.1097/CCM.0000000000002095)
Supplement: Supplementary file 1 [file ccm-45-282-s001.docx]

**Online Supplement for Vitamin D deficiency in human and murine sepsis.**

Dhruv Parekh^1,3*^, Jaimin Patel^1,4*^, Aaron Scott^1^, Sian Lax^1^, Rachel CA Dancer^1^, Vijay D’Souza^1^, Hannah Greenwood^1^, William D Fraser^4^, Fang Gao^1,4^, Elizabeth Sapey^1^, Gavin D Perkins^3,4^ , David R Thickett^1^

1. Centre for Translational Inflammation Research, Institute of Inflammation and Aging, University of Birmingham, UK
2. Norwich Medical School, University of East Anglia, UK.
3. Warwick Clinical Trials Unit, Warwick Medical School, University of Warwick, UK
4. Heart of England NHS Foundation Trust, Bordesley Green, Birmingham, B9 5SS

Corresponding author: Professor David Thickett [d.thickett@bham.ac.uk](mailto:d.thickett@bham.ac.uk). Telephone sec+44 121 371 4841 Fax +44 121 627 8245. These authors denoted by * are joint first authors.

**Detailed Methods:**

**Study Participants**:

Patients were recruited from the acute medical admissions units (AMU) and ICUs at two University Hospitals (Heart of England NHS Foundation Trust and the University Hospital Birmingham NHS Foundation Trust) between September 2012 and October 2014.

**Ethical Approvals**: All patients and healthy volunteers in the studies reported provided informed written consent. In circumstances where patients were unable to provide consent a legal representative (personal or designated consultee) provided assent. Retrospective consent was sought where possible, when patients regained the ability to consent. This study received the appropriate ethical and local approvals (Regional Ethics Committee references: 11/SC/0356 & 11/YH/0270)

**Inclusion criteria:** age greater than 18 years; documented new proven or suspected infection, and the presence of any two of the signs and symptoms of infection (WCC > 11 or < 4 × 10^9^/L, temperature > 38°C or < 36°C, heart rate > 90/bpm, or respiratory rate > 20/minute) for less than 24 hours. Patients were categorised as sepsis or severe sepsis according to the presence of one or more organ failure (1).

**Exclusion criteria:** Age under 18, recent chemotherapy, chronic steroid use or use of other immunosuppressants.

Data were extracted from the medical records, laboratory reports and intensive care charts. Acute Physiology and Chronic Health Evaluation II (APACHE II) and serial organ failure assessment (SOFA) scores were recorded at baseline. Appropriate microbiological samples were sent for culture the site of infection and pathogen. Collection was guided by clinical condition. Full blood count, plasma urea and electrolytes liver function tests, coagulation studies and arterial blood gas analysis were recorded. We followed all participants up to 12 months after enrolment and collected data for length of hospital stay, 30 day, 90 day and 1-year mortality.

**Laboratory Methods:**

**Plasma preparation:**

Blood was collected into 7ml Lithium Heparin vacutainer® tubes (Becton Dickinson Ltd, Oxford, UK). These were then transported on ice to the laboratory for processing and analysis. Whole blood was placed in a centrifuge pre-chilled to 4^o^c and spun at 560g for 10 minutes. The supernatant (plasma) was then aspirated and aliquoted into cryovials and stored at -80^O^C as described previously (2).

**Vitamin D status**: 25(OH)D_3_ was measured by tandem mass spectronomy using appropriate DEQAS control(3). Definition of vitamin D status is controversial, with different figures used throughout the literature but for this study we have considered plasma 25(OH)D_3_ concentrations below 50 nmol/l (20 ng/mL) as deficient. 25(OH)D_3_ concnetration between 50 and 75 nmol/l (30 ng/mL) as insufficient, with concentrations above 75 nmol/l (30ng/mL) designated sufficient levels (4). 1,25(OH)_2_D3 concentrations were measured by ELISA (Immunodiagnostic Systems, UK) and expressed as pg/ml.

**Animal MATERIALS AND METHODS**

Several studies have suggest low vitamin D levels in sepsis patients but it is unclear whether this represents a biological marker of overall health status or a mechanistically important risk factor. We therefore undertook animal experiments in mice that were made vitamin D deficient by diet using the caecal and ligation puncture (CLP) model of bacterial sepsis, and the well characterised lung injury model, IT- LPS (5, 6).

**Induction of vitamin D deficiency:**

Male wild-type (WT) C57Bl/6 mice were obtained from Harlan UK Limited, Oxford, UK and maintained at the Biomedical Servicing Unit (BMSU), Birmingham University, UK. Once weaned, vitamin D deficiency was induced in WT pups by feeding them a vitamin D deficient chow (TD 89123, Harlan, USA) or normal chow for 6 weeks. All animals were housed in cages of 6 in a 12-hour light and 12-hour dark cycle with food and water provided *ad libitum*. All procedures were performed in accordance to UK laws and with approval from the local animal ethics committee.

**Cecal ligation and puncture:**

Pre medication was given in the form of subcutaneous buprenorphine (Temgesic® 0.1mg/kg body weight) 15-30 minutes prior to the procedure. Animals were anaesthetised with isoflurane gas (5%) with oxygen for induction and maintenance (1-3%) anaesthesia. All surgery was performed with aseptic techniques. Midline laparotomy was performed followed by exposure of the caecum, ligation of the lower 30% with 2.0 Ethilon^TM^ nylon suture (Ethicon, UK) and single through-and-through puncture of ligated caecum with a 19G microlance needle (BD, Becton, Dickinson and Company). A small amount of faeces was then gently expressed by compressing the ligated caecum with forceps prior to being placed back into the abdomen and closed with 6.0 Vicryl^®^ (Ethicon, UK) followed by skin closure with 4.0 Prolene^®^ (Ethicon, UK).. Surgery was performed on heated tables set at 37.5^o^C. All animals were recovered in heat boxes and recovery incubators until sacrificed. Postoperative resuscitation was with 0.5mls of fluid (Hartman’s solution, Aqupharm 11®) and a further dose of buprenorphine. Animals were reassessed 6 hours post-operatively and given a further 0.5mls of fluid and sacrificed at 16 hours post surgery. Sham surgery was identical except for the lack of ligation and puncture of the caecum after exteriorisation from the abdomen.

**Intratracheal (IT) LPS challenge:**

IT instillations were performed as previously described(5). Briefly, mice were anaesthetised using intraperitoneal injections of metetomidine (60 mg/kg) and ketamine (10 mg/kg) and a fine polyethylene catheter (external diameter 0.61 mm and internal diameter 0.28 mm) passed into the trachea via the mouth under direct visualisation of the vocal cords. Fifty micrograms LPS (Source Biosciences, UK) in 50 µL sterile PBS or PBS alone were instilled. Mice were given 0.1 mL atipamezole to reverse the metetomidine and hydrated with two 0.5 mL saline subcutaneous injections, one immediately post IT and another 6 h later.

**Sample collection and processing**

At 16 hours post CLP animals were deeply anaesthetised with 5% isoflurane. Cardiac puncture was performed and death confirmed. Immediate post-mortem peritoneal lavage fluid (PLF) was collected by instilling 1.0ml of PBS/EDTA into the right and left upper quadrant of the abdomen and aspirating in both lower quadrants. Bronchoalveolar lavage fluif (BALF) was collected by opening the chest cavity and neck, dissecting down, manipulating the trachea and inserting a fine bore polythene tubing to the level of just above the carina. Lungs were then lavaged twice with 0.6mls of PBS/EDTA (200nM). Examination of the caecum was carried out to confirm necrosis and colour of liver recorded to ensure adequate sepsis was induced. Cardiac blood was centrifuged at 13,500RPM in a micro-centrifuge for 10 minutes. Prior to centrifugation 15ul of whole blood was taken for bacterial culture. The sera layer was aspirated and stored at -20^o^C. PLF and BALF was centrifuged at 400g for 10minutes. The supernatant was aspirated and stored at -20^O^C.

**Murine vitamin D and Calcium status**

For murine studies, calcium and alkaline phosphatase (ALP) were measured by colorimetric assay (Abcam, UK) and parathyroid hormone (PTH) by ELISA (Immutopics International). 25(OH)D3 was measured by ELISA (ImmunDiagnostik, Germany). All assays performed as per manufacturer’s protocols.

**Bacterial culture**

Whole blood, PLF and BALF were diluted accordingly and incubated at 37^o^C in pre-prepared Lysogeny [broth](https://en.wikipedia.org/wiki/Broth) (LB-Lennox) agar plates and incubated at 37^o^C for 24 hours. Colony forming units (CFU) were counted and CFU/ml calculated from the original dilutions.

**Cell analysis**

BALF and PLF were analysed for cellular inflammation by cell count, neutrophil count and number of apoptotic neutrophils by cell surface staining and flow cytometry (CyAn™ ADP, Beckman Coulter). The following markers and strategy was used for cellular identification; macrophages (CD11b^high^ F4/80^high^ Gr1^low^); neutrophils (CD11b^high^ F4/80^low^ Gr1^high^); apoptosis assessed with annexin V and sytox staining.

**Protein assay**

A Bio-rad® simple colorimetric assay was used to measure total protein in sera, BALF and PLF. Bovine serum albumin (BSA) standards of known concentration were made up to 10ul and plated along with 10ul of sample. Bio-Rad^®^ solution was diluted 1 in 5 with distilled water and 200ul added to each well which turns blue on contact with protein. Absorbance was measured immediately at 595nm (Synergy 2 Gen 5^TM^, Biotek). Protein permeability index (PPI) was calculated as a ratio fluid protein: plasma protein.

**ELISA assays**

Murine BAL RAGE was measured by a commercially purchased ELISA (R&D systems, Abingdon, UK).

**Ex-vivo macrophage phagocytosis assay**

In brief extracted peritoneal cells underwent red cell lysis using Gibco ACK lysing buffer. Cells were washed with PBS/BSA 2% at 400g for 4 minutes and plated into a microplate and pHrodo ®labelled E.coli bioparticles added and incubated for 30 mins at 37^0^C. After incubation cells were washed once more and stained with the cell surface markers described above. Macrophage phagocytosis of the particle results in acidic pH change resulting in fluorescence which was measured by flow cytometry (CyAn™ ADP, Beckman Coulter).

**Cathelicidin related antimicrobial peptide (CRAMP) assay.**

Total CRAMP levels were measured in sera, BALF and PLF by an indirect ELISA. CRAMP standards of known concentrations and samples were made up to 50 µl in serum free media and incubated in Greiner high binding plates (Sigma-Aldrich) overnight at 37 ^0^C in an unsealed plate to allow adherence. Plates were washed 3 x with PBST (0.05%) and blocked in PBST containing 1% BSA for 1 hour at room temperature with gentle agitation. Rabbit anti-CRAMP antibody (Innovagen) was diluted 1:2500 in PBST (0.05%) and 100 µl added to each well for 2 hours at room temperature. Plates were again washed 3 x with PBST (0.05%) and 100 µl of goat anti-rabbit HRP antibody diluted 1:2500 in PBST (0.05%) added per well for 1 hour at room temperature.

Plates were washed with PBST (0.05%) and HRP activity was measured by the addition of TMB (100 μl per well). Plates were incubated with substrate for 10 minutes in the dark before the reaction was stopped by addition of 100 µl of stop solution (1M HCl). Absorbance values were obtained at 450 nm within 10 minutes, (Synergy 2 Gen 5^TM^, Biotek). Sample concentrations were then extrapolated from the appropriate standard curve. CRAMP levels were corrected per mg of protein and represented as ng/mg.

**Statistics:** Data were analyzed using SPSS for Windows 16.0 (SPSS, Inc., Chicago, IL). Data were tested for normality and analyzed by unpaired *t* tests or Mann-Whitney U test. Data are expressed as mean (SD) unless otherwise indicated. A Chi squared or Fisher's exact test was used to compare proportions. A p value of 0.05 was considered significant.

**RESULTS**

**25(OH)D_3_ concentrations are lower in patients with severe sepsis compared to sepsis and healthy controls.**

None of severe sepsis patients had sufficient (> 75 nmol/L) 25(OH)D concentrations with 36 (87.5 %) being vitamin D deficient (< 50nmol/L) and 5 (12.5%) being insufficient (50-75 nmol/L). Of the sepsis patients 1 (5%) had sufficient 25(OH)D concentrations whilst 13 (62%) were deficient and 6 (30%) were insufficient. 6 (30%) of healthy controls had sufficient 25(OH)D concentrations, 8 (40%) were deficient and 6 (30%) were insufficient

**
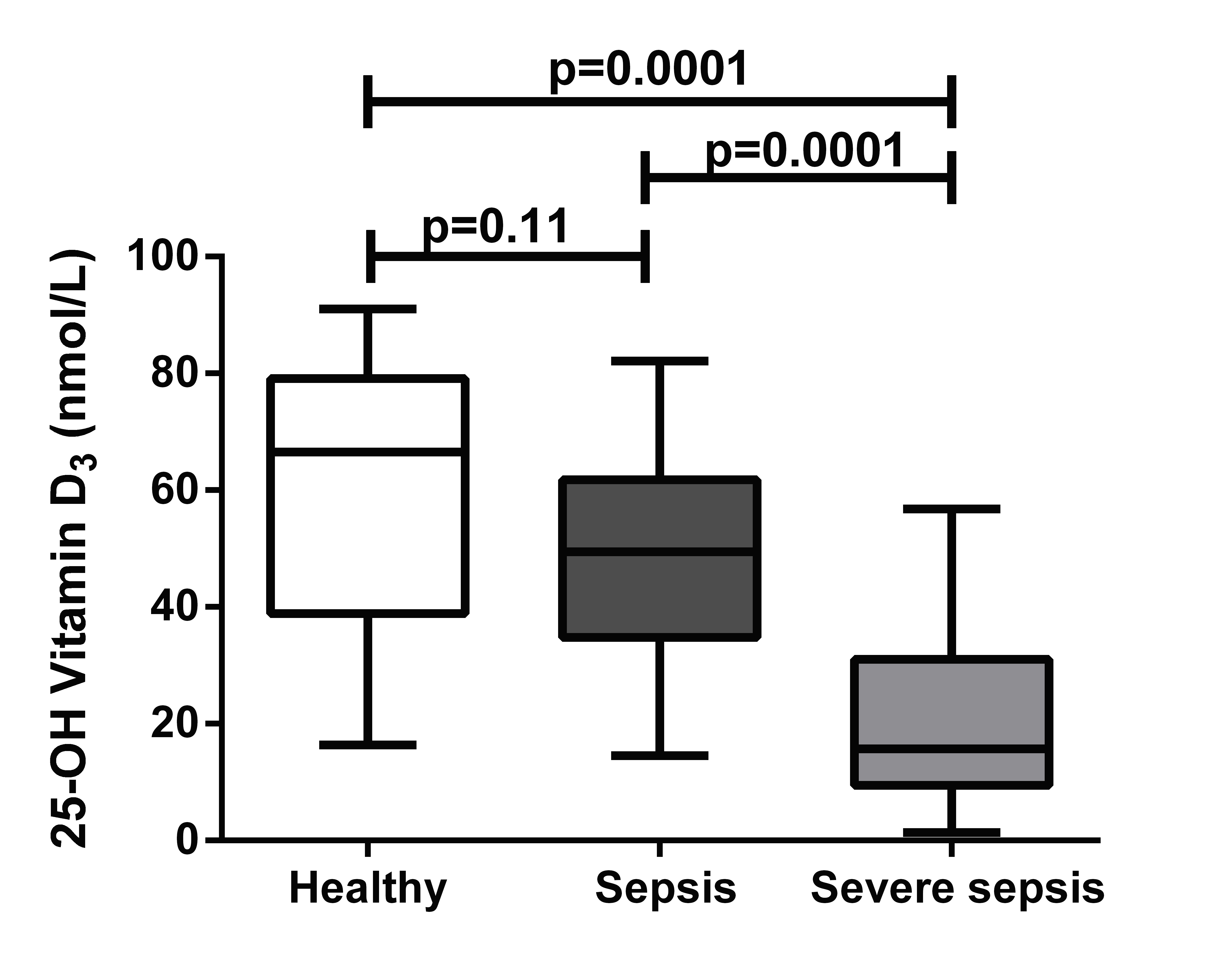
**

**Online supplement figure 1: Comparison of plasma 25(OH)D_3_ between patient groups and volunteers.** Box and whisker plots represent median with Tukey’s distribution.

**25(OH) D_3_ concentrations are lower in sepsis patients with positive microbial cultures.**

**
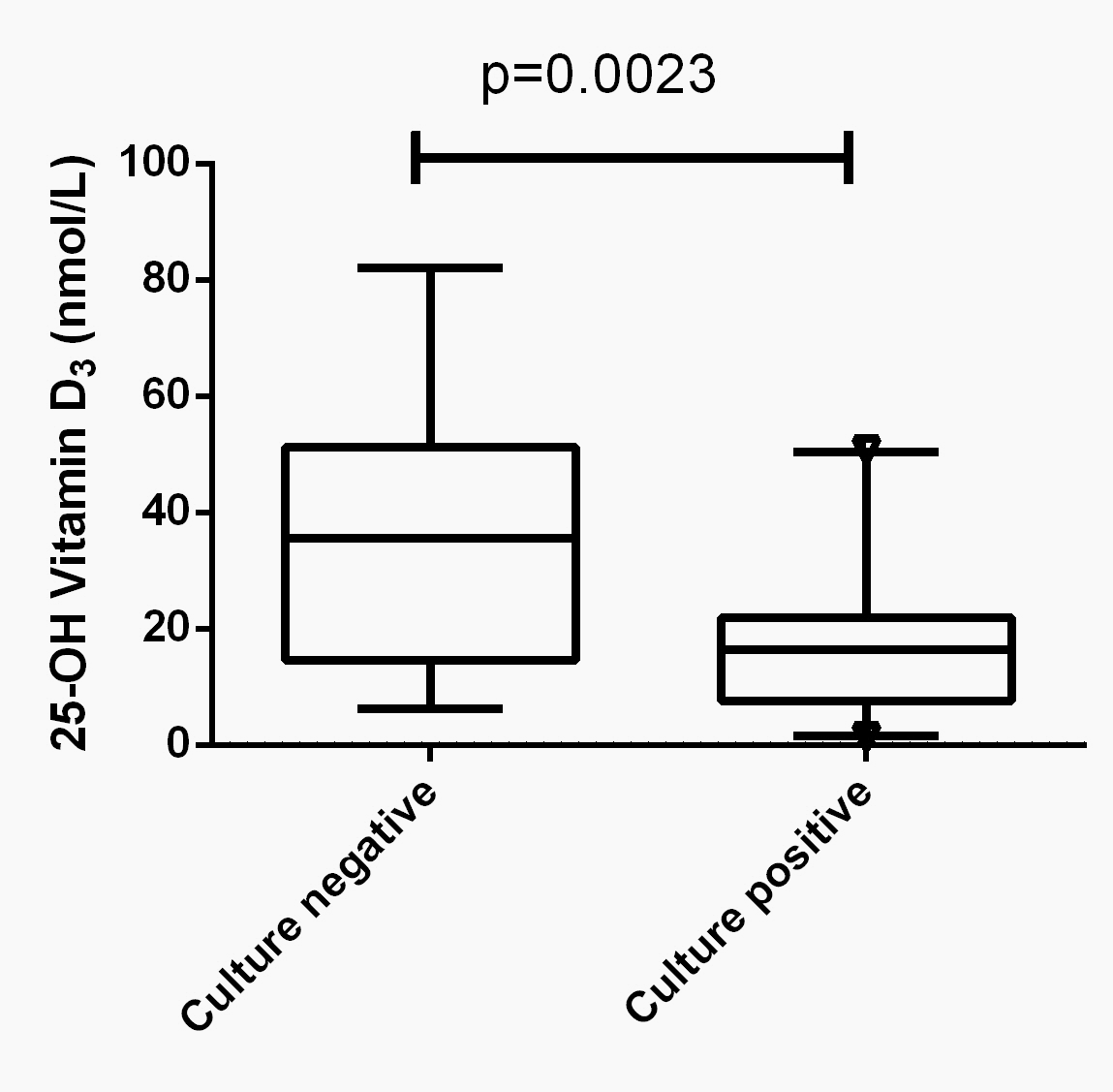
**

**Online supplement figure 2: Plasma 25(OH)D_3_ between patients who were culture positive (n=22) versus culture negative (n=39).** Box and whisker plots with median and Tukey’s distribution.

**VDD was successfully established in wild type (WT) C57BL/6 mice fed a deficient diet compared to a vitamin D sufficient (VDS) diet** (online supplement table 1).

|  | VDD  n = 8 | VDS  n = 8 | p-value |
| --- | --- | --- | --- |
| 25(OH)D_3_ (nmol/L) | 9.0  (5.0-11.7) | 50.4  (48.1-51.9) | 0.0003 |
| 1,25(OH)_2_D_3_(pg/ml) | 13.4  (7.3-18.9) | 150.5  (125.0-175.3) | 0.002 |
| Calcium (mM) | 3.4  (3.1-4.1) | 3.3  (3.1-3.4) | 0.69 |
| PTH (pg/ml) | 82.3  (57.0-91.6) | 112.5  (62.1-139.6) | 0.470 |
| ALP Activity (μmol/min/ml) | 6.1  (5.1-7.1) | 8.0  (7.1-9.1) | 0.0154 |

Online supplement table 1: Effects of vitamin D deficient diet after 6 weeks on circulating vitamin D levels. Data presented as medians (interquartile range); Mann-Whitney U statistical test. VDD (vitamin D deficient); VDS (vitamin D sufficient); PTH (parathyroid hormone); ALP (alkaline phosphatase).

**CLP does not induce alveolar neutrophilia but does increase protein permeability index, which is more pronounced in vitamin D deficiency.**

**
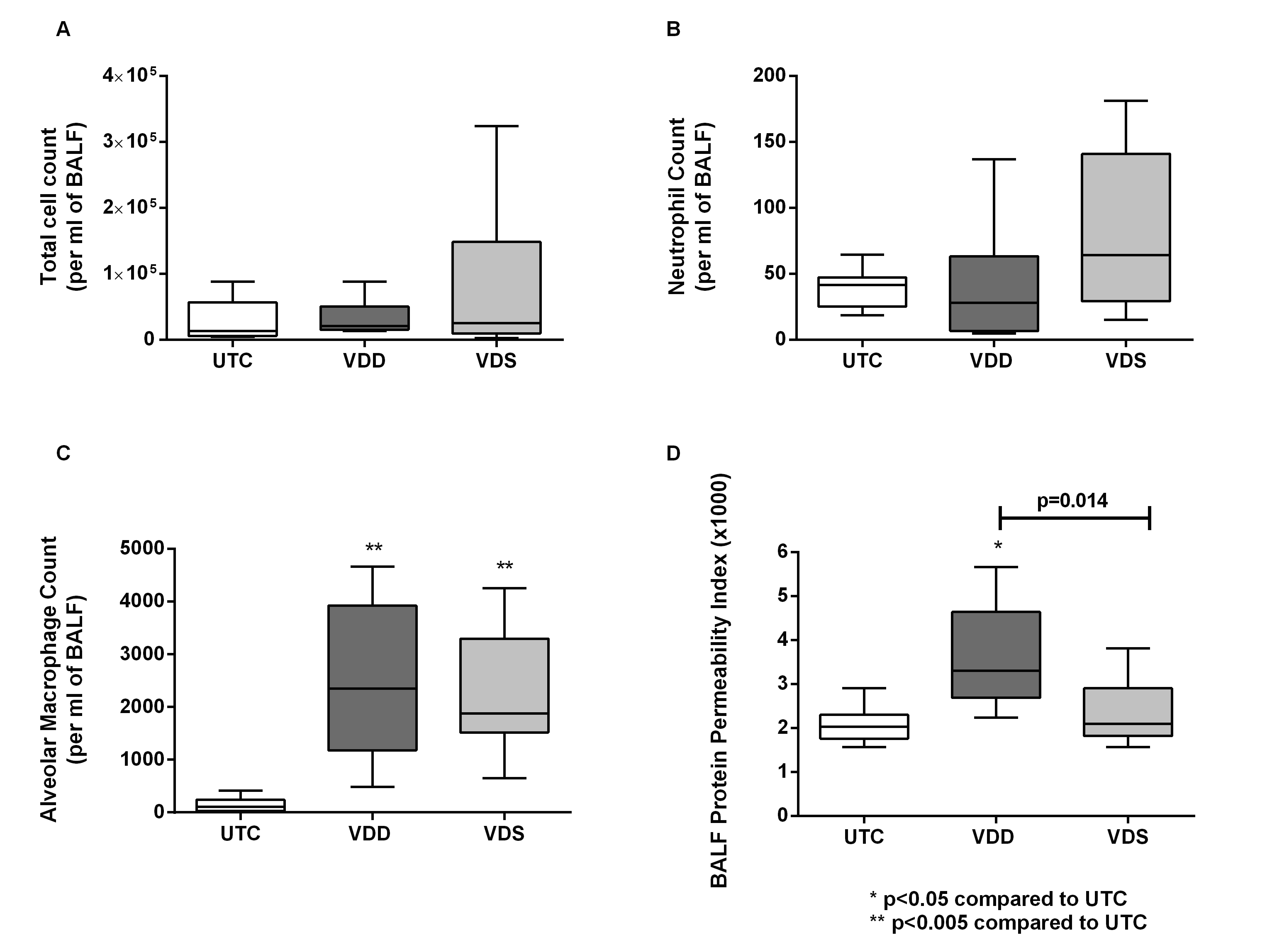
**

**Online supplement figure 3: BALF cellular recruitment 16 hours post CLP** Box and whisker plots with median and Tukey’s distribution. **A**. total cell count p=0.57; **B**. neutrophil count p= 0.33 and **C**. alveolar macrophage count p=0.001. **D** BALF protein permeability index (p=0.014). VDD (vitamin D deficient) n=8; VDS (vitamin D sufficient) n=7; UTC (untreated WT control) n= 10.

**Vitamin D deficiency is associated with increased cellular inflammation in the peritoneum.**


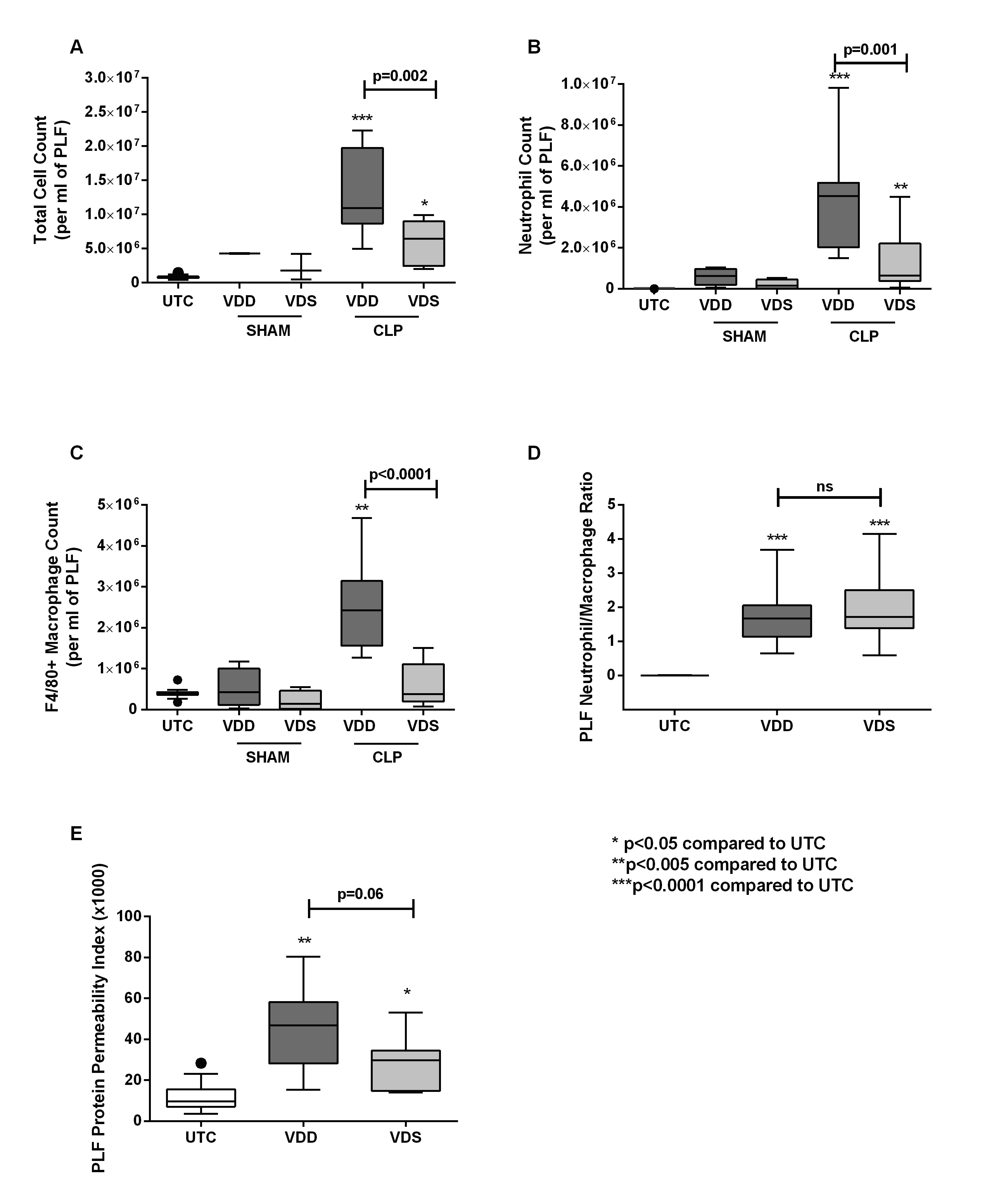


**Online supplement figure 4: PLF cellular recruitment 16 hours post CLP.** Box and whisker plots with median and Tukey’s distribution. **A**. total cell count p<0.0001; **B**. neutrophil count p<0.0001 and **C**. F4/80 + macrophage count p<0.0001). **D** PLF neutrophil/macrophage ratio. **E** PLF protein permeability index. VDD (vitamin D deficient) n=12; VDS (vitamin D sufficient) n=11; UTC untreated WT control n=10. Sham experiments did not significantly increase cellular infiltration.

**Effect of IP Cholecalciferol (Vigantol™) on vitamin D levels in dietary deficient mice:**

In preliminary experiments we tested the effect of 1500 IU of IP cholecalciferol liquid upon vitamin D levels in VDD mice 48 hours after administration. IP cholecalciferol was effective in restoring the plasma 25(OH)D_3_ levels of VDD deficient mice to those of WT mice (online supplement figure 5)

.


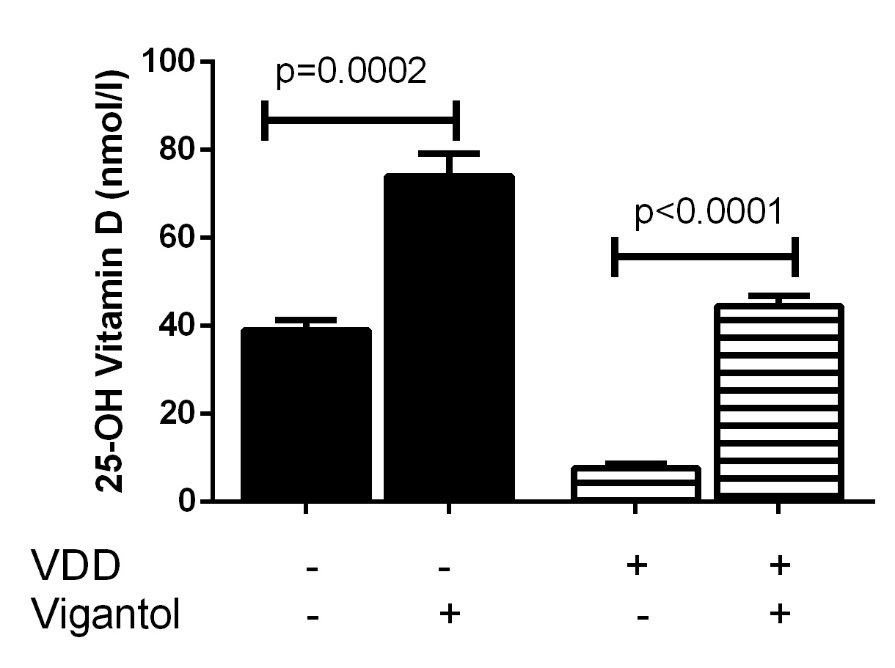


**Online supplement figure 5:** Effect of 1500 IU of IP cholecalciferol upon vitamin d levels of mice given standard diet as opposed to vitamin D deficient chow (VDD). Plasma levels of 25(OH)D_3_ were measured 48 hours after IP administration. N= 6 in each group.

References:

1. Patel JM, Snaith C, Thickett DR, Linhartova L, Melody T, Hawkey P, Barnett AH, Jones A, Hong T, Cooke MW, Perkins GD, Gao F. Randomized double-blind placebo-controlled trial of 40 mg/day of atorvastatin in reducing the severity of sepsis in ward patients (ASEPSIS Trial). *Crit Care* 2012; 16: R231.

2. Richter AG, Stockley RA, Harper L, Thickett DR. Pulmonary infection in Wegener granulomatosis and idiopathic pulmonary fibrosis. *Thorax* 2009; 64: 692-697.

3. Owens DJ, Webber D, Impey SG, Tang J, Donovan TF, Fraser WD, Morton JP, Close GL. Vitamin D supplementation does not improve human skeletal muscle contractile properties in insufficient young males. *Eur J Appl Physiol* 2014; 114: 1309-1320.

4. Marcinowska-Suchowierska E, Walicka M, Talalaj M, Horst-Sikorska W, Ignaszak-Szczepaniak M, Sewerynek E. Vitamin D supplementation in adults - guidelines. *Endokrynol Pol* 2010; 61: 723-729.

5. Lax S, Wilson MR, Takata M, Thickett DR. Using a non-invasive assessment of lung injury in a murine model of acute lung injury. *BMJ open respiratory research* 2014; 1: e000014.

6. Dancer RC, Parekh D, Lax S, D'Souza V, Zheng S, Bassford CR, Park D, Bartis DG, Mahida R, Turner AM, Sapey E, Wei W, Naidu B, Stewart PM, Fraser WD, Christopher KB, Cooper MS, Gao F, Sansom DM, Martineau AR, Perkins GD, Thickett DR. Vitamin D deficiency contributes directly to the acute respiratory distress syndrome (ARDS). *Thorax* 2015; 70: 617-624.
